# Crystal structure of a novel homodimeric l‐ribulose 3‐epimerase from *Methylomonus* sp.

**DOI:** 10.1002/2211-5463.13159

**Published:** 2021-05-01

**Authors:** Hiromi Yoshida, Akihide Yoshihara, Shiro Kato, Susumu Mochizuki, Kazuya Akimitsu, Ken Izumori, Shigehiro Kamitori

**Affiliations:** ^1^ Life Science Research Center and Faculty of Medicine Kagawa University Kita Japan; ^2^ International Institute of Rare Sugar Research and Education Kagawa University Kita Japan; ^3^ Faculty of Agriculture Kagawa University Kita Japan

**Keywords:** d‐allulose, d‐allulose 3‐epimerase, l‐ribulose 3 epimerase, rare sugar, X‐ray structure, β/α‐barrel

## Abstract

d‐Allulose has potential as a low‐calorie sweetener which can suppress fat accumulation. Several enzymes capable of d‐allulose production have been isolated, including d‐tagatose 3‐epimerases. Here, we report the isolation of a novel protein from *Methylomonas* sp. expected to be a putative enzyme based on sequence similarity to ketose 3‐epimerase. The synthesized gene encoding the deduced ketose 3‐epimerase was expressed as a recombinant enzyme in *Escherichia* *coli*, and it exhibited the highest enzymatic activity toward l‐ribulose, followed by d‐ribulose and d‐allulose. The X‐ray structure analysis of l‐ribulose 3‐epimerase from *Methylomonas* sp. (MetLRE) revealed a homodimeric enzyme, the first reported structure of dimeric l‐ribulose 3‐epimerase. The monomeric structure of MetLRE is similar to that of homotetrameric l‐ribulose 3‐epimerases, but the short C‐terminal α‐helix of MetLRE is unique and different from those of known l‐ribulose 3 epimerases. The length of the C‐terminal α‐helix was thought to be involved in tetramerization and increasing stability; however, the addition of residues to MetLRE at the C terminus did not lead to tetramer formation. MetLRE is the first dimeric l‐ribulose 3‐epimerase identified to exhibit high relative activity toward d‐allulose.

## Graphical abstract

d‐Allulose has potential as a low‐calorie sweetener which can suppress fat accumulation. The enzymes capable of d‐allulose production have attracted attention. Here, we report the X‐ray structure of a novel l‐ribulose 3‐epimerase from *Methylomonas* sp. (MetLRE) which also has enzymatic activity towards d‐allulose, showing that MetLRE is the first dimeric l‐ribulose 3‐epimerase identified to exhibit high relative activity toward d‐allulose.

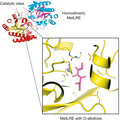

**Abbreviations**
AgDAE: d‐allulose 3‐epimerase from *Arthrobacter globiformis*
AtDAE: d‐allulose 3‐epimerase from *Agrobacterium tumefaciens*
CcDAE: d‐allulose 3‐epimerase from *Clostridium cellulolyticum*
DAE: d‐allulose 3‐epimerase
his_MetLRE long2: his_MetLRE with nine additional residues (TAIKTIELH) at the C terminus and with three mutations (Y9I, Y37F, Y286L)
his_MetLRE: N‐terminal his‐tagged MetLRE
his_MetLRE3: his_MetLRE with three mutations (Y9I, Y37F, and Y286L)
LRE: l‐ribulose 3‐epimerase
MetLRE: l‐ribulose 3‐epimerase from *Methylomonas* sp.
MlLRE: l‐ribulose 3‐epimerase from *Mesorhizobium loti*
PcDTE: d‐tagatose 3‐epimerases from *Pseudomonas cichorii*

d‐Allulose (alternative name d‐psicose) is a rare sugar, and its physiological functions, such as altering the blood glucose level, suppressing fat accumulation, and use as a low‐calorie sweetener [1, 2, 3, 4, 5, 6, 7, 8, 9], have been focused on as a promising functional food ingredient. In Japan, a low‐calorie syrup containing d‐allulose, Rare Sugar Sweet^®^ (Matsutani Chemical Industry Co., Ltd., Hyogo, Japan) [10], is commercially available and its labeling as a functional food was recently approved. In addition, the U.S. Food and Drug Administration (FDA) stated that ‘it allows the low‐calorie sweetener allulose to be excluded from total and added sugar counts on Nutrition and Supplement Facts labels when used as an ingredient’ (FDA In Brief, April, 2019).

Several enzymes capable of d‐allulose production have been reported, including d‐tagatose 3‐epimerases from *Pseudomonas cichorii* [11, 12, 13], *Rhodobacter sphaeroides* [14], *Sinorhizobium* sp. [15] and *Caballeronia fortuita* [16], d‐allulose 3‐epimerases from *Agrobacterium tumefaciens* [17], *Agrobacterium* sp. [18], *Clostridium bolteae* [19], *Clostridium cellulolyticum* [20], *Clostridium scindens* [21], *Clostridium* sp. [22], *Desmospora* sp. [23], *Dorea* sp. [24], *Rhodopirellula baltica* [25], *Ruminococcus* sp. [26], *Staphylococcus aureus* [27] and *Treponema primitia* [28], and ketose 3‐epimerase from *Arthrobacter globiformis* [29]. Currently, only a few crystal structures are available from the Protein Data Bank (PDB). There are only four available structures of the potential enzymes for d‐allulose production from the abundant sugar d‐fructose: *P*. *cichorii* d‐tagatose 3‐epimerase (PcDTE, 290 amino acids, homodimer) [30], d‐allulose 3‐epimerases from *A. tumefaciens* (AtDAE, 289 amino acids, homotetramer) [17], *C. cellulolyticum* (CcDAE, 293 amino acids, homotetramer) [31], and *A. globiformis* ketose 3‐epimerase (289 amino acids, homotetramer) [32], which was originally reported as d‐allulose 3‐epimerase (AgDAE) due to its similar substrate specificity to the known d‐allulose 3‐epimerases. AgDAE exhibits the highest activity toward l‐ribulose, and its structure is similar to that of l‐ribulose 3‐epimerase from *Mesorhizobium loti* (MlLRE, 297 amino acids, homotetramer) [33], although MlLRE has little activity toward d‐allulose [34].

Based on the sequence similarity with these potential enzymes by BLAST search, we selected a putative protein from *Methylomonas* sp. DH‐1 (NCBI, WP_064020855.1, 286 a.a.) as a putative sugar isomerase/epimerase.

In this study, using a synthesized gene encoding the putative sugar isomerase/epimerase from *Methylomonas* sp., the expression, characterization, crystallization, and structure determination of the putative protein were performed. In the first screening, enzymatic activity toward d‐allulose was confirmed and the recombinant enzyme from *Methylomonas* sp. was suspected to be d‐allulose 3‐epimerase. In further investigation of its substrate specificity, the enzyme exhibited the highest enzymatic activity toward l‐ribulose, followed by d‐ribulose and d‐allulose (Fig. 1). Therefore, it was considered to be l‐ribulose 3‐epimerase (LRE), but it also had relatively high enzymatic activity toward d‐allulose. X‐ray structure analysis of the LRE from *Methylomonas* sp. DH‐1 (MetLRE) revealed a homodimer different from previously reported homotetrameric LREs, including AgDAE, and the most structurally similar protein to MetLRE is AgDAE, which has a long C‐terminal α‐helix. The crystal structure of MetLRE is the first known homodimeric l‐ribulose 3‐epimerase with a short C‐terminal α‐helix. The effects of the length of the C‐terminal α‐helix on forming a homotetramer were discussed.

**Fig. 1:**
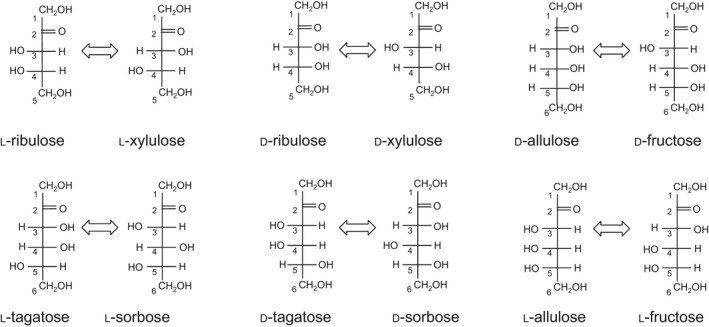
Potential substrates of MetLRE presented in a Fischer projection. The epimerization reaction is shown by the arrow.

## Results and Discussion

In this study, the crystal structures of C‐terminal his‐tagged MetLRE alone and in complexes with d‐fructose, d‐allulose, and l‐tagatose were determined at resolutions of 2.08, 1.80, 1.80, and 1.70 Å, respectively. In addition, the crystal structures of N‐terminal his‐tagged MetLRE in complex with d‐allulose and modified MetLRE with additional C‐terminal residues in complex with d‐fructose were determined at resolutions of 2.05 and 1.58 Å, respectively.

### Overall structure of MetLRE_his in complex with d‐fructose

**Fig. 2:**
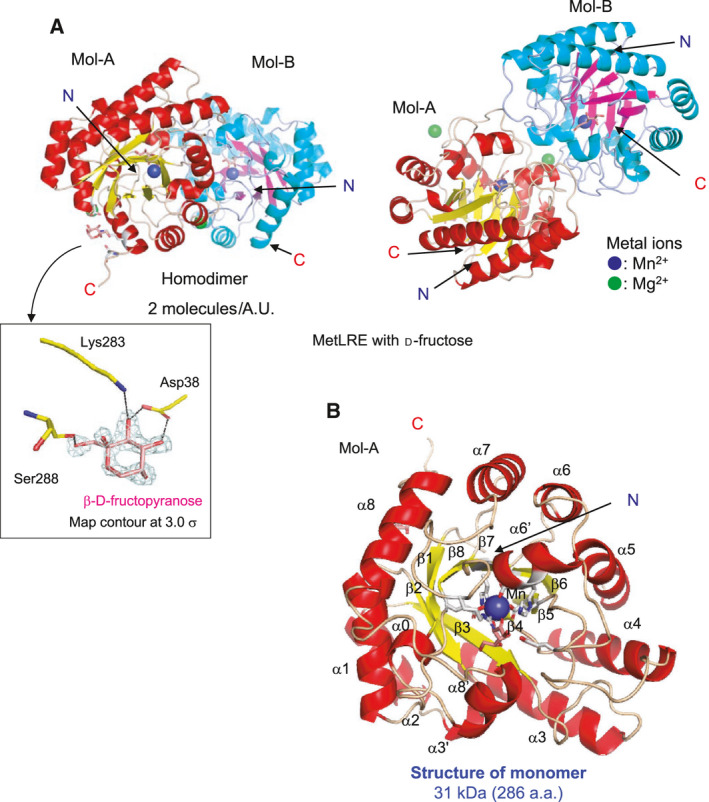
Overall structure of MetLRE in complex with d‐fructose. (A) Dimeric structure of MetLRE in complex with d‐fructose. Front view (left) and top view (right) are shown with the bound metal ions (blue and green spheres) and d‐fructose (pink stick). N and C indicate the N terminus and C‐terminus, respectively. A bound β‐d‐fructopyranose around the C‐terminal region in Mol‐A is shown with SA‐omit maps at the contour level of 3.0 σ. (B) The subunit structure of MetLRE. The residues involved in metal coordination and the bound d‐fructose are shown as white and pink stick models, respectively.

MetLRE_his in complex with d‐fructose was crystallized in the orthorhombic space group *P*2_1_ *2*_1_2_1_ with two molecules in an asymmetric unit. MetLRE forms a homodimer (Mol‐A and Mol‐B) and the catalytic sites of the molecules are located on the same side (Fig. 2A, top view). There are four bound metal ions in the structure, a metal ion at the catalytic site in each molecule, and the others on the surface of the protein molecules. The former metals were found in all X‐ray structures and considered to be manganese that was added in the medium for protein expression in *Escherichia coli* cells (1 mm MnCl_2_), whereas the bound metal ions on the surface of the protein molecules are not found in all X‐ray structures and were considered to originate from a reservoir solution containing magnesium (0.2 m MgCl_2_) used for crystallization. The metal ions were checked by CheckMyMetal web server (https://cmm.minorlab.org/) [35]. The catalytic metals were acceptable as Mn. The bound metals on the surface of the protein molecules were suggested to be Ca as an alternative metal to Mg; however, the B‐factor and geometry of Mg were acceptable or borderline, and the bound metals were refined as Mg considering the concentration of MgCl_2_ in the reservoir solution.

In Mol‐A, the C terminus was refined to His293 including a linker and part of the His‐tag, and the C terminus of Mol‐B was refined to Gly287, which is the beginning of a linker before the His‐tag. As bound β‐d‐fructopyranose was found around the C‐terminal region of Mol‐A, the His‐tag region was able to be refined in Mol‐A. The carbonyl oxygen of Ser288 in the linker forms a hydrogen bond with O2 of β‐d‐fructopyranose, Lys283, and Asp38 interact with O3, and Asp38 forms a hydrogen bond with O4 of the bound β‐d‐fructopyranose. In the structure of MetLRE/d‐fructose, Mol‐A and Mol‐B are almost identical, with an r.m.s.d. of 0.58 Å for the corresponding Cα atoms. Hereafter, the description of the monomeric structure of MetLRE is concentrated on Mol‐A (unless stated otherwise).

The monomeric structure has twelve α‐helices and eight β‐strands, forming a (β/α)_8_ barrel fold with four additional short α‐helices (α0, α3′, α6′, and α8′) (Fig. 2B). There is a metal ion at the center of the barrel, which is coordinated by Glu152, Asp185, His211, and Glu246 to form the catalytic site and bind the substrate. The bound metal ion at the catalytic site was refined as Mn^2+^ because MetLRE was expressed in cells in medium containing 1 mm MnCl_2_, and strong electron density maps for a metal ion at the catalytic site were also observed from the crystal grown in the metal‐free reservoir solution.

**Fig. 3:**
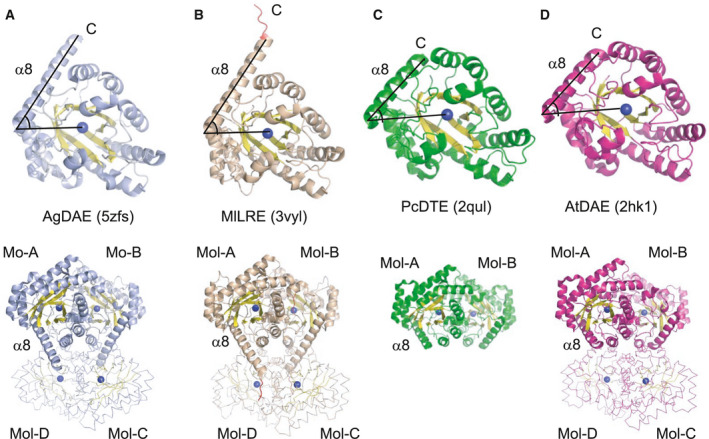
Overall structure of the structurally similar enzymes. The monomeric structure (up) and front view (down) of the overall structure of structurally similar enzymes are represented. (A) *Arthrobacter globiformis* d‐allulose 3‐epimerase (AgDAE), (B) *Mesorhizobium loti* l‐ribulose 3‐epimerase (MlLRE), (C) *Pseudomonas cichorii* d‐tagatose 3‐epimerase (PcDTE), and (D) *Agrobacterium* *tumefaciens* d‐allulose 3‐epimerase (AtDAE). For each overall structure, the dimer form is shown as a cartoon model and the other dimer of the tetrameric form is shown as a ribbon model. The C‐terminal α‐helix is labeled as α8.

The most structurally similar protein was ketose 3‐epimerase from *Ar. globiformis* (previously known as AgDAE, l‐ribulose 3‐epimerase, PDB code 5ZFS, [32]), with 54% identity, 1.6 Å r.m.s.d. and 45.6 *Z*‐score by Dali search [36, 37]. Other similar proteins were l‐ribulose 3‐epimerase from *M. loti* (MlLRE, 3VYL, 40%, 1.3 Å, 42.5, [33]), d‐tagatose 3‐epimerase from *P. cichorii* (PcDTE, 2QUL, 30%, 1.7 Å, 37.3, [30]), and d‐allulose 3‐epimerase from *A. tumefaciens* (AtDAE, 2HK1, 28%, 1.8 Å, 36.8, [17]). The monomeric structures of these proteins are compared in Fig. 3. The monomeric structure of these proteins adopts a (β/α)_8_ barrel fold, and a metal ion is bound at the center of the barrel. Although the features of LREs are a long C‐terminal helix (α8, 26–29 residues) and formation of stable homotetramers, MetLRE has a shorter C‐terminal helix (α8, 16 residues) than LREs and forms a homodimer like homodimeric PcDTE. A similar feature of MetLRE with LRE was the angle of the catalytic metal–N terminus of α8–C terminus of α8 (metal‐α8N‐α8C), which was 54°, being between MlLRE (58°) and AgDAE (52°), and different from PcDTE (49°) and AtDAE (43°) (Fig. 3 and Table 1).

**Table 1:** Features of monomeric structures of comparable enzymes.

Enzymes	Number of residues	Oligomer formation	Identity (%)	r.m.s.d.	*Z*‐score	α8 residues and angle (metal‐α8N‐α8C)
MetLRE	286	Dimer	100	1.0		S271‐Y286 (16)^a^, 54°
AgDAE (LRE)	289	Tetramer	55	1.6	45.6	D264‐H289 (26), 52°
MlLRE	297	Tetramer	40	1.3	42.5	D265‐A293 (29), 58°
PcDTE	290	Dimer	30	1.7	37.9	T270‐A290 (21), 49°
AtDAE	289	Tetramer	28	1.8	36.8	D268‐G288 (21), 43°

^a^ The number of the residues is presented in the parenthesis.

### Protein analysis and enzymatic activity

The molecular size of MetLRE was estimated to be 53 057 Da by size exclusion chromatography. As the molecular weight of the monomeric MetLRE was approximately 32 kDa by SDS/PAGE (calculated MW is 31.3 kDa based on the amino acid residues), MetLRE likely forms a rigid homodimer in solution considering its crystal structure.

**Fig. 4:**
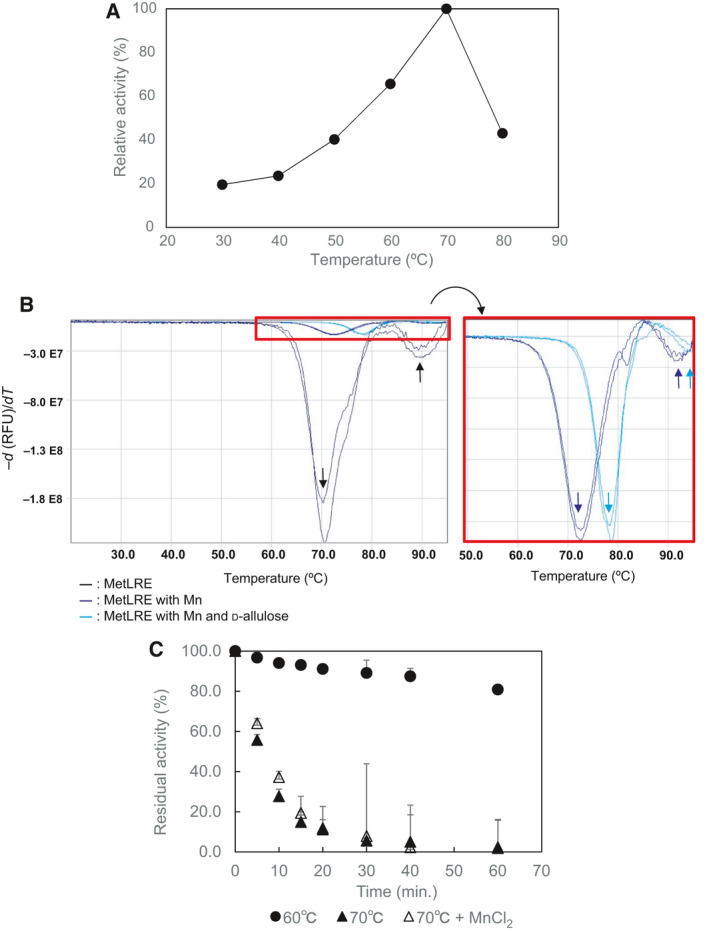
Effects of temperature on the activity of MetLRE. (A) Temperature dependence, (B) melting temperature analysis, and (C) thermal stability analysis. The error bars represent SD.

After we confirmed enzymatic activity of the recombinant enzyme toward d‐allulose, the optimum temperature of the enzyme was found to be 70 °C, as shown in Fig. 4A. Compared with the optimum temperatures of AgDAE (70 °C, [29]), MlLRE (60 °C, [34]), PcDTE (60 °C, [12]), AtDAE (50 °C, [17]), and CcDAE (60 °C, [22]), this homodimeric enzyme belongs to the group having a higher optimum temperature.

In a thermal shift assay, two Tm values (Tm_1_ = 70.5 ± 0.1 °C, Tm_2_ = 89.7 ± 0.3 °C) of MetLRE were found in the absence of Mn and d‐allulose (Fig. 4B, black line). MetLRE is likely unfolded in two steps. In addition, the Tm values of MetLRE in the presence of Mn (blue line) and in the presence of Mn and d‐allulose (cyan line) were 72.5 ± 0.03 °C (Tm_1__Mn) and 92.3 ± 0.4 °C (Tm_2__Mn), and 78.5 ± 0.1 °C (Tm_1__MnA) and 94.4 ± 0.2 °C (Tm_2__MnA), respectively. The shift in Tm values suggests that MetLRE is stabilized in the presence of Mn and d‐allulose.

As additional thermal stability evaluation of MetLRE, the residual activity was measured in the presence and absence of MnCl_2_ at 70 °C (Fig. 4C). The residual activity of MetLRE with Mn was slightly higher than that of MetLRE alone for initial 15 min, but each residual activity was less than 40% after 10 min incubation. The half‐life time of MetLRE in the absence of MnCl_2_ was calculated as 208 ± 2.0 min at 60 °C (Fig. 4C).

Considering the structural similarity with d‐allulose 3‐epimerases and l‐ribulose 3‐epimerases, we investigated the enzymatic activity toward d,l‐ketohexoses and d,l‐ketopentoses at 70 °C (Fig. 1, Table 2). The highest activity of the enzyme was detected toward l‐ribulose. Compared with the highest activity of 82.1 U·mg^−1^ to l‐ribulose (100%), the recombinant enzyme exhibited enzymatic activities of 66.4 U·mg^−1^ for d‐ribulose (80.9%), 53.3 U·mg^−1^ for d‐allulose (65.0%), 47.8 U·mg^−1^ for d‐fructose (58.3%), 25.6 U·mg^−1^ for l‐xylulose (31.2%), and 22.0 U·mg^−1^ for l‐tagatose (26.8%). The *K*_m_ and *k*_cat_ values of the enzyme toward l‐ribulose were 22.4 ± 3.4 mm and 4923 ± 346 min^−1^, and those toward d‐allulose were 27.8 ± 2.2 mm and 4103 ± 179 min^−1^, respectively. This enzyme is a l‐ribulose 3‐epimerase, but it has relatively high activity toward d‐allulose.

**Table 2:** Substrate specificity of MetLRE and comparison with that of other LREs. The enzyme activity of MetLRE_his was measured after incubation in a buffer (50 mm Tris/HCl pH 7.5) containing 1 mm MnCl_2_ and each substrate at 100 mm at 70 °C for 10 min. Each measurement was performed in triplicate.

Substrate	MetLRE		AgDAE^a^		MlLRE^b^	
	Specific activity (U·mg^−1^)	Relative activity (%)	Specific activity (U·mg^−1^)	Relative activity (%)	Specific activity (U·mg^−1^)	Relative activity (%)
l‐Ribulose	82.1 ± 2.3	100 ± 2.7	121.3	100	230	100
d‐Ribulose	66.4 ± 2.0	80.9 ± 2.4	21.4	17.6	46	20
d‐Allulose	53.3 ± 0.4	65.0 ± 0.5	77.0	63.5	5.3	2.3
d‐Fructose	47.8 ± 0.1	58.3 ± 0.2	–	–	4.0	1.7
l‐Xylulose	25.6 ± 0.1	31.2 ± 0.2	18.0	14.8	96	42
l‐Tagatose	22.0 ± 0.3	26.8 ± 0.3	–	–	5.5	2.4
d‐Xylulose	14.2 ± 0.3	17.3 ± 0.3	10.2	8.4	12	5.4
d‐Tagatose	13.6 ± 0.2	16.6 ± 0.2	–	–	55	24
l‐Sorbose	7.5 ± 0.01	9.2 ± 0.01	–	–	Trace	Trace
d‐Sorbose	6.8 ± 0.07	8.3 ± 0.08	–	–	14	6.0
l‐Allulose	3.7 ± 0.05	4.6 ± 0.06	–	–	31	14
l‐Fructose	0.81 ± 0.02	0.99 ± 0.02	–	–	10	4.1

^a^ Yoshihara *et al*. [29], Yoshida *et al*. [32].

^b^ Uechi *et al*. [34].

### Ligand‐binding structure at the catalytic site

#### Ligand‐free and d‐fructose

**Fig. 5:**
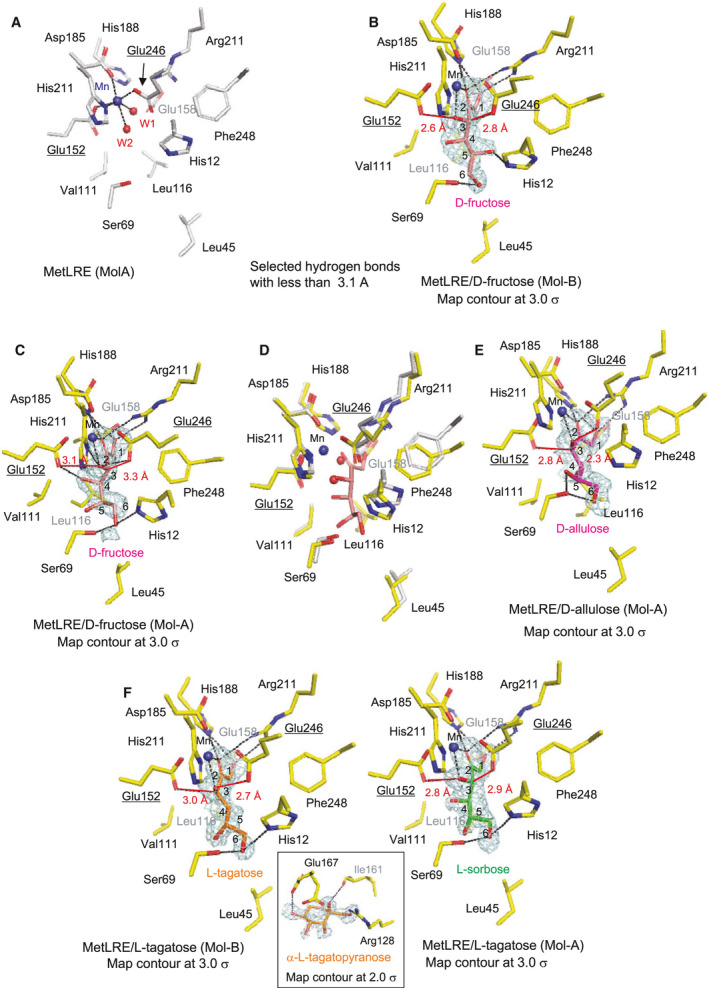
Structures of the catalytic site of MetLRE in complexes with ligands. Catalytic sites of (A) ligand‐free MetLRE (Mol‐A), (B) MetLRE/d‐fructose (Mol‐B), (C) MetLRE/d‐fructose (Mol‐A), (D) superimposed model of (C) on (A), (E) MetLRE/d‐allulose (Mol‐A), and (F) MetLRE/l‐tagatose [Mol‐B (left) and Mol‐A (right)] and a bound α‐l‐tagatopyranose in Mol‐B (center). Each ligand is illustrated as a stick model, d‐fructose (B–D, pink), d‐allulose (E, magenta), l‐tagatose (F, orange), and l‐sorbose (F, green). A metal ion and water molecules are shown in blue and red spheres, respectively. Selected interactions among amino acid residues, ligands, water molecules, and a metal ion are indicated by dotted lines. The bound ligands in MetLRE are shown with the SA‐omit maps at 2.0–3.0 σ contours.

The structure of the catalytic site in ligand‐free MetLRE is shown in Fig. 5A. The r.m.s.d. for the Cα atoms between Mol‐A and Mol‐B is 0.20 Å (the refined regions: Ala2‐Gly287 in both molecules). A metal ion is coordinated by two catalytic glutamate residues (Glu152 and Glu246), Asp185, His211 and two water molecules (W1 and W2). In the structure of Mol‐B with bound d‐fructose of MetLRE/d‐fructose (Fig. 5B), O1 of d‐fructose forms hydrogen bonds with Glu158, His188, and Arg211, and O2 and O3 coordinate with a metal ion instead of W1 and W2 in the ligand‐free structure. O2 of d‐fructose also forms a hydrogen bond with His188 and Arg211. O3 is recognized by catalytic Glu152. O5 and O6 of d‐fructose are recognized by His12 and Ser69 through hydrogen bonds, with a distance of 2.8 and 2.7 Å, respectively. In the structure of Mol‐A (Fig. 5C), O4, O5, and O6 of d‐fructose were slightly shifted rotating around the C3‐C4 axis. O4 forms a hydrogen bond with Glu152, with a distance of 2.6 Å, and O6 interacts with His12 and Ser69, with a distance of 2.8 and 2.9 Å, respectively.

The two catalytic glutamates (Glu152 and Glu246) face the substrate in the structure of MetLRE/d‐fructose, as observed in the complex structure of PcDTE with d‐fructose [30]. While one catalytic Glu152 forms a hydrogen bond with O3, with a distance of 2.6 Å, the other catalytic Glu246 is directed to the hydrogen atom of C3 with a distance of 2.8 Å, in Mol‐B. Glu246 can remove a proton at C3 of the substrate in the epimerization reaction of MetLRE, as suggested in PcDTE [30, 38]. Thus, MetLRE can produce d‐allulose from d‐fructose through the C3–O3 proton exchange mechanism via a *cis*‐enediolate intermediate. In Mol‐A of MetLRE/d‐fructose, Glu152 can interact with O3, with a distance of 3.1 Å, but the distance between Glu246 and C3 of d‐fructose is 3.3 Å. As Glu152 prefers to form a hydrogen bond with O4 (2.6 Å) in Mol‐A, MetLRE was unable to catalyze the epimerization of the substrate in this situation. As the electron density maps of d‐fructose in Mol‐A were slightly weaker than those in Mol‐B, a bound form of MetLRE may exist.

On comparison of the active site structures of ligand‐free MetLRE and Mol‐A of MetLRE/d‐fructose (Fig. 5D), His12 and Phe248 approach d‐fructose in the structure of MetLRE/d‐fructose. As the large shift in His12 and Phe248 was not found in Mol‐B of MetLRE/d‐fructose or in other complex structures of MetLRE with substrates, the movement of a loop region (β8‐α8′) including Phe248 may affect the proper position of substrate binding and slightly rotate the bound substrate around the C3‐C4 axis. In other conditions, the movement of the loop region leads to proper positioning of substrate binding.

#### d‐Allulose

In the structure of MetLRE in complex with d‐allulose, Mol‐A and Mol‐B are almost identical (0.29 Å r.m.s.d., Ala2‐Gly287 in both molecules). The bound d‐allulose was found in the catalytic site of both Mol‐A and Mol‐B. The structure of the catalytic site with bound d‐allulose in Mol‐A is shown in Fig. 5E. O2 and O3 of d‐allulose coordinate with the metal ion, forming hydrogen bonds with His188 and Glu246, with a distance of 2.9 and 2.3 Å, respectively. Glu152 is directed to the hydrogen atom attached to C3 with a distance of 2.8 Å. These distances are suitable for the C3–O3 proton exchange mechanism, suggesting that d‐allulose is also recognized as a substrate of MetLRE. Although O4 of d‐allulose faces the hydrophobic pocket (formed by Leu45, Val111, Leu116, and Leu257) and cannot form a hydrogen bond with any residue, O5 forms a hydrogen bond with Ser69 (2.9 Å), and O6 interacts with Ser69 with a distance of 2.7 Å.

#### l‐Tagatose

The structures of Mol‐A and Mol‐B in MetLRE with bound l‐tagatose are also almost identical (0.26 Å r.m.s.d., Ala2‐Gly287 in both molecules). The bound l‐tagatose was found in the catalytic site of Mol‐B, and a moderately bound α‐l‐tagatopyranose was found on the surface of Mol‐B (O1, O2, and O5 of α‐l‐tagatopyranose interact with the carbonyl oxygen of Ile161, Arg128, and Glu167, respectively).

The structure of the catalytic site with bound l‐tagatose in Mol‐B of MetLRE/l‐tagatose is shown in Fig. 5F. O2 and O3 of l‐tagatose coordinate with the metal ion, forming hydrogen bonds with His188 and Glu246, with a distance of 3.1 and 2.7 Å, respectively. Glu152 is directed to the hydrogen atom attached to C3 with a distance of 3.0 Å. O4 and O5 are directed to the hydrophobic pocket and do not form a hydrogen bond with the enzyme. Instead, O6 is recognized by His12 and Ser69, with a distance of 2.9 and 2.5 Å, respectively. In Mol‐A of MetLRE/l‐tagatose, l‐sorbose (C3 epimer of l‐tagatose) was bound in the catalytic site based on the electron density maps. Glu152 forms a hydrogen bond with O3 of l‐sorbose (2.8 Å), and the distance between Glu246 and C3 of l‐sorbose is 2.9 Å. The bound l‐tagatose was epimerized at the position of C3 and converted to l‐sorbose. O6 also forms hydrogen bonds with His12 and Ser69, with a distance of 2.8 and 2.6 Å, respectively.

**Fig. 6:**
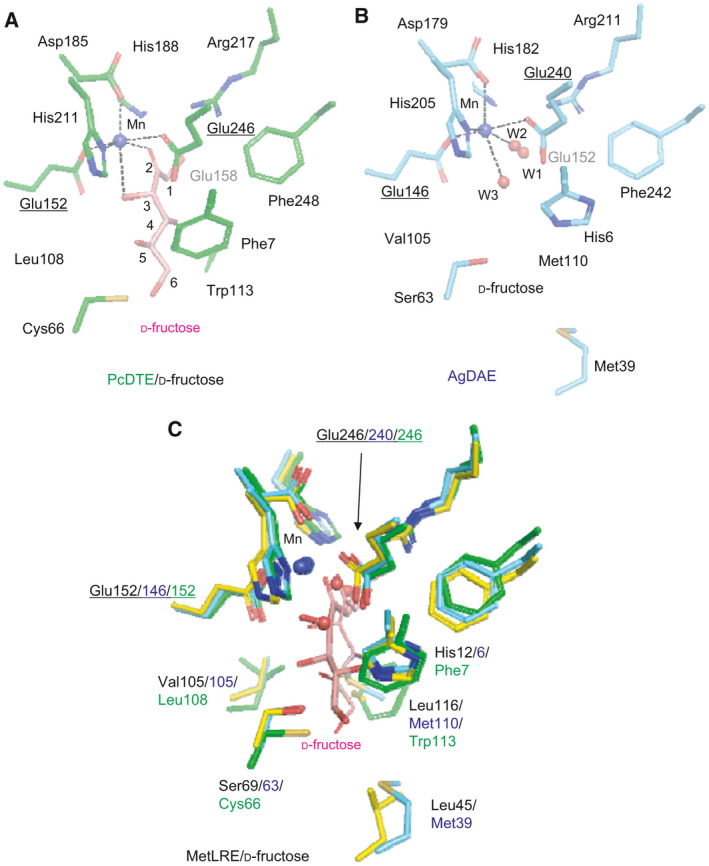
Comparison of the structure of the catalytic site of MetLRE with the related enzymes PcDTE and AgDAE. Catalytic sites of (A) PcDTE/d‐fructose (PDB code: 2QUN), (B) AgDAE (5ZFS), and (C) superimposed model of (A) and (B) on Fig. 5B. The bound d‐fructose of PcDTE/d‐fructose is represented as a line model in (C). Each ligand is illustrated as a stick model, d‐fructose (A, C, pink). A metal ion and water molecules are shown in blue and red spheres, respectively. Selected interactions among amino acid residues, ligands, water molecules, and a metal ion are indicated by dotted lines.

In the previously reported structure of PcDTE/d‐fructose (Fig. 6A), PcDTE formed strong interactions with the 1, 2, and 3 positions of the substrate with strong electron density, and a relatively weak interaction occurred at the 4, 5, and 6 positions of the substrate with poor electron density. The structures of MetLRE in the complexes with the above substrates with strong electron density maps suggested stronger interaction with the substrate at the 4, 5, and 6 positions. Recognition of the substrates in the catalytic structure of MetLRE is consistent with the enzymatic activities toward ketohexoses.

On comparison of the active site structure of MetLRE/d‐fructose (Fig. 5B) with those of PcDTE/d‐fructose (Fig. 6A) and AgDAE (Fig. 6B), all metal‐binding sites of PcDTE (Glu152, Asp185, His211, and Glu246) and AgDAE (Glu146, Asp179, His205, and Glu240) are conserved in MetLRE (Glu152, Asp185, His211, and Glu246), as shown in Figs 5B and 6A–C. As the two catalytic glutamate residues are also conserved in these enzymes, including AtDAE and CcDAE, a common catalytic reaction mechanism was proposed.

In this study, we were unable to determine the complex structure with l‐ribulose and d‐ribulose and cannot explain their mode of binding; however, MetLRE preferably binds ketopentoses with the same orientation of the hydroxyl groups at C3 and C4 positions in the Fischer projection (*cis*‐configuration) (Fig. 1, Table 2). The orientation of the hydroxyl group at C5 of ketohexoses together with the hydroxyl group at C6 affects the enzymatic activity toward ketohexoses by forming hydrogen bonds with MetLRE. On comparison between d‐allulose and l‐tagatose (or d‐tagatose and l‐allulose), the orientation of the hydroxyl group at C5 influenced the enzymatic activity. Therefore, the hydroxyl group at C5 of ketopentoses may also affect the activity, and MetLRE prefers to bind the l‐form of ketopentoses according to its enzymatic activity. As an example of the structure in complex with ketohexose harboring a different orientation of the hydroxyl groups at C3 and C4 positions (*trans*‐configuration), two binding forms were observed in the complex structure of MetLRE/d‐fructose (Fig. 5B,C). In one form (Fig. 5C), O4 of d‐fructose can form a hydrogen bond with catalytic residue Glu152 and likely influences the catalytic activity. This may be a reason for why MetLRE prefers substrates with hydroxyl groups at C3 and C4 in a *cis*‐configuration and exhibits relatively high activity toward d‐allulose. However, as the recognition mechanism of substrates with a ring‐form and the ring‐opening mechanism of MetLRE are unknown, further studies are needed to elucidate the substrate specificity in detail.

Moreover, we found that some residues approach the substrate to stabilize the active site, as shown in Fig. 5D, and the Tm shifted in the presence of d‐allulose (Fig. 4B). MetLRE changes to fit the substrate, which was not observed in our previous study on the homodimer enzyme PcDTE. Therefore, some residues, including His12, Leu45, and Phe248, may approach closer to ketopentoses such as l‐ribulose and d‐ribulose by forming hydrogen bonds with the substrate and increasing the hydrophobicity at the opposite face.

Of note, this enzyme exhibited the highest activity toward l‐ribulose, followed by d‐ribulose, at the optimum temperature (70 °C). At 70 °C, the residues recognizing substrates, such as His12, Ser69, and Phe248, may be more flexible in the active site and recognize l‐ribulose as a preferable substrate.

### Effects of the length of the C‐terminal α‐helix of MetLRE

**Fig. 7:**
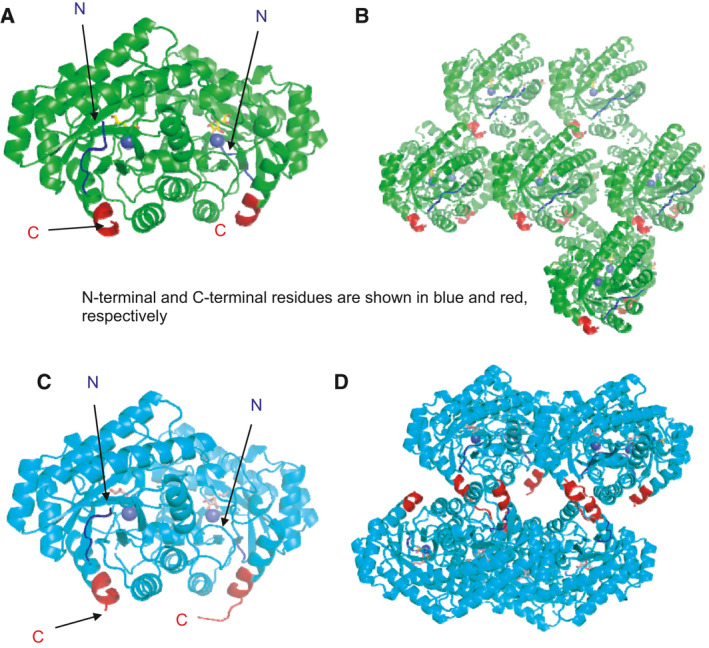
Structures of N‐terminal his‐tagged MetLRE (his_MetLRE) and modified MetLRE with the C‐terminal tail (his_MetLRElong2), and their crystal packing. N‐ and C‐terminal regions are shown in blue and red, respectively. A metal ion is represented as a blue sphere. (A) Front view of the overall structure of his_MetLRE that forms a homodimer. (B) Crystal packing of his_MetLRE. Each dimer aligns in the same direction in a crystal. (C) Front view of the overall structure of his_MetLRElong2 that forms a homodimer. (D) Crystal packing of his_MetLRElong2. Dimers align in the same direction in one line and other dimers align in the opposite direction in another line. Two dimers face each other, but they cannot form a homotetramer.

MetLRE was found from the genome database by amino acid similarity search with AgDAE and was thought to form a homotetramer such as AgDAE from a similar enzyme family. However, MetLRE formed a homodimer and has a shorter C‐terminal helix (α8) than AgDAE, and its dimer form is similar to that of PcDTE with a relatively short α8. To eliminate the influence of the C‐terminal His‐tag, a new expression vector for MetLRE using the plasmid pET15b was constructed. A recombinant N‐terminal His‐tagged MetLRE (his_MetLRE) was prepared, and its crystal structure was determined. His_MetLRE in complex with d‐allulose was obtained in the triclinic space group *P*1 (*a* = 51.02, *b* = 81.05, *c* = 106.13, α = 99.30, β = 101.50, γ = 87.24) with six molecules in an asymmetric unit. His_MetLRE also forms a homodimer similar to C‐terminal his‐tagged MetLRE, but does not form a tetramer by symmetry operation (Fig. 7A,B). Although the electron density maps of the His‐tagged N terminus were not fully visible, a part of the additional N‐terminal region did not form a secondary structure and was not affected by oligomer formation. We next tried to make MetLRE form a homotetramer by elongating the C‐terminal α8 with additional residues. Based on the tetramer form of AgDAE, N‐terminal his_tagged Metlong2 (his_MetLRElong2) was prepared with nine residues (TAIKTIELH) added at the C terminus of MetLRE and three residues (Y9I, Y37F, and Y286L) were replaced to increase intramolecular interactions and eliminate the influence of intermolecular steric hindrance in the expected tetramer formation. Using the recombinant protein of his_MetLRElong2, the crystal structure of his_MetLRElong2 in complex with d‐fructose was determined. The crystal was obtained in the triclinic space group *P*1 (*a* = 80.43, *b* = 92.77, *c* = 93.78, α = 87.67, β = 98.64, γ = 115.60) with eight molecules in an asymmetric unit. His_MetLRElong2 forms a homodimer. The visible additional C‐terminal region did not elongate the α‐helix, and his_MetLRElong2 did not form a tetramer by symmetry operation or PISA analysis [39]. However, the additional C‐terminal tail affected the crystal packing because his_MetLRE and his_MetLRElong2 were crystallized under the same conditions using the same reservoir solution but their crystal packing differed. The dimer form of his_MetLRElong2 aligns face to face with the side of the C‐terminal region, although they did not contact each other to form a tetramer (Fig. 7C,D). In addition, we investigated the effects of the additional C‐terminal residues on the thermal stability of his_MetLRElong2 and compared his_MetLRE and the newly constructed his_MetLRE3 that contains triple mutations (Y9I, Y37F, and Y286L) (Table 3). The optimum temperature of all was 80 °C in the presence of MnCl_2_ (his_MetLRElong2: 68.1 ± 1.0 U·mg^−1^; his_MetLRE3: 119.7 ± 2.3 U·mg^−1^; his_MetLRE: 71.5 ± 1.6 U·mg^−1^). The relative activity of his_MetLRElong2 at 70 °C (87.1%) was slightly higher than that of his_MetLRE (81.8%), and the relative activities of his_MetLRElong2 at 50 °C (40.6%) and 60 °C (55.0%) were almost similar to those of his_MetLRE. As the relative activities of his_MetLRE3 were slightly higher than those of his_MetLRE, the triple mutations (Y9I, Y37F, and Y286L) affected the thermal stability by increasing the intramolecular interactions, but the additional C‐terminal residues in the loop region did not affect the thermal stability, because the region was unable to form a secondary structure and the length of the α‐helix was not extended, precluding tetramer formation. In a homotetramer, a smaller angle is related to a shorter C‐terminal α‐helix, and a larger angle is related to a longer C‐terminal α‐helix (Table 1). They are correlated: (the angle of metal‐α8N‐α8c) = 1.8673 × (the number of residues) + 3.6939 with *R*_2_ = 0.9992. In this case, MetLRE requires 27 residues for the C‐terminal α‐helix to form a tetramer. Thus, nine additional residues were insufficient, and an entire α‐helix consisting of 27 residues may be needed.

**Table 3:** Thermal stability of his_MetLRElong2, his_MetLRE3, and his_MetLRE. The enzyme activity toward 100 mm d‐allulose was measured using 50 mm Tris/HCl pH 7.5 containing 1 mm MnCl_2_ after a 10 min incubation at each temperature. his_MetLRE3 contains triple mutations (Y9I, Y37F, and Y286L).

Temperature (°C)	His_MetLRElong2		His_MetLRE3		His_MetLRE	
	Activity (U·mg^−1^)	Relative activity (%)	Activity (U·mg^−1^)	Relative activity (%)	Activity (U·mg^−1^)	Relative activity (%)
50	27.7 ± 0.6	40.6 ± 0.9	49.7 ± 1.2	41.5 ± 1.0	26.9 ± 1.5	37.7 ± 2.1
60	37.5 ± 0.6	55.0 ± 0.9	72.1 ± 1.8	60.2 ± 1.5	41.3 ± 1.5	57.7 ± 2.1
70	59.3 ± 2.0	87.1 ± 2.9	101.1 ± 2.6	84.5 ± 2.2	58.5 ± 0.1	81.8 ± 0.1
80	68.1 ± 1.0	100 ± 1.5	119.7 ± 2.3	100 ± 1.9	71.5 ± 1.6	100 ± 2.2

Recently, Shin *et al*. [40] reported an enzyme from *Thermotoga maritima* to be a novel type of l‐ribulose 3‐epimerase, which was found as a putative d‐tagatose 3‐epimerase‐related protein produced from the TM0416 gene (TM0416, 271 amino acids, homodimer). The X‐ray structure of TM0416 was previously reported by Sakuraba *et al*. [41], but its substrate was unknown. Shin *et al*. screened the enzyme as a ketose 3‐epimerase, and it exhibited the highest enzymatic activity toward d‐erythrose (18.7 U·mg^−1^, 100%), l‐ribulose (5.0 U·mg^−1^, 27.0%), d‐ribulose (4.4 U·mg^−1^, 24.0%), and d‐threose (4.1 U·mg^−1^, 22.0%). The activities toward d‐allulose (0.1 U·mg^−1^, 0.5%) and d‐fructose (0.1 U·mg^−1^, 0.5%) were low. As for d‐erythrose and d‐threose (aldotetrose), TM0146 does not catalyze the epimerization at C‐3 position for these aldotetrose, but it does catalyze the aldose–ketose isomerization of d‐erythrose and d‐threose to convert d‐erythrulose. The highest *k*_cat_ values of TM0416 toward l‐xylulose, d‐erythrose, l‐ribulose, and d‐ribulose were 86.3, 74.8, 3.7, and 37.9 min^−1^, respectively. Considering the *k*_cat_/*K*_m_ (l‐xylulose: 0.19 mm ^−1^·min^−1^; d‐erythrose: 0.17 mm ^−1^·min^−1^; l‐ribulose: 0.40 mm ^−1^·min^−1^; d‐erythrose: 0.11 mm ^−1^·min^−1^), they concluded that TM0416 can be classified as a novel type of l‐ribulose 3‐epimerase with strong isomerization activity for aldotetrose.

According to Dali search, the similarity of MetLRE to TM0416 is 29%, 2.0 Å r.m.s.d., 31.0. The monomeric structure of TM0416 is also similar to a (β/α)_8_ barrel fold and C‐terminal α‐helix (α8: G250‐K268, 19 residues), but the region between β8 and α8 is shorter (E243‐G249, 7 residues) in TM0416 than in MetLRE (E246‐D270, 25 residues), other LREs (24 residues), and DAEs (24 residues). As this region covers the active site like a lid in LREs and DAEs, and substrate binding sites around ketohexose at O4, O5, and O6 are also different in TM0416, MetLRE has different substrate specificity from TM0416 and is a novel homodimeric l‐ribulose 3‐epimerase.

## Conclusion

In this study, we expressed a novel homodimeric l‐ribulose 3‐epimerase from *Methylomonas* sp. and determined its X‐ray structure. As the structure contained a unique short C‐terminal α‐helix compared to the known structures of the structurally similar homotetrameric l‐ribulose 3‐epimerases and d‐allulose 3‐epimerases, we attempted to form tetrameric MetLRE by adding C‐terminal residues, but we were unable to obtain the tetrameric form of MetLRE. As the dimeric form of MetLRE is relatively stable compared to the optimum temperature of homodimeric PcDTE (60 °C, [12]), MetLRE may be an optimized dimeric enzyme and a new type of l‐ribulose 3‐epimerase with relatively high activity toward d‐allulose.

## Materials and methods

### Cloning and expression

An expression vector was constructed using the vector pQE60 (QIAGEN, Hilden, Germany) and a synthesized gene encoding the putative sugar isomerase/epimerase from *Methylomonas* sp. DH‐1 (NCBI, WP_064020855.1). The synthesized gene was amplified using the following primers: 5′‐GAGAAATTAACCATGGCCTTTCCGAAACGCCTG‐3′ (forward primer) and 5′‐GATGGTGATGAGATCCGTACCGTTGTTTGATGAAT‐3′ (reverse primer) and mixed with the digested pQE60 by *Noc*I and *Bgl*II for In‐Fusion reaction using the In‐Fusion HD Cloning Kit (TaKaRa Bio Inc., Shiga, Japan). Recombinant MetLRE was expressed in *E. coli* JM109 containing the resultant plasmid pQEMetLRE as a His‐tagged protein at the C terminus (MetLRE_his). For the expression of N‐terminal His‐tagged MetLRE (his_MetLRE), a pair of two primers, 5′‐CGCGCGGCAGCCATAGCATGGCCTTTCCGAAAC‐3′ (forward) and 5′‐GGATCCTCGAGCATATTAGTACCGTTGTTTGATGA‐3′ (reverse), and the expression vector pET15b (Novagen, Madison, WI, USA) digested with *Nde*I were used to construct pETMetLRE. The recombinant his_MetLRE was expressed in *E. coli* BL21(DE3).

For the expression of N‐terminal His‐tagged MetLRElong2 (his_MetLRElong2), another synthesized gene encoding MetLRE with mutations of Y9I, Y37F, and Y286L, and nine additional residues (TAIKTIELH) at the C terminus was used as template to form a homotetramer. A pair of primers, 5′‐CGCGCGGCAGCCATAGCATGGCGTTTCCGAAAC‐3′ (forward primer) and 5′‐GATCCTCGAGCATATTAATGGAGTTCAATGGTCTT‐3′ (reverse primer), and the expression vector pET15b digested with *Nde*I were used to construct pETMetLRElong2. The recombinant his_MetLRElong2 was expressed in *E. coli* BL21(DE3).

### Protein preparation

*Escherichia coli* cells expressing each recombinant MetLRE were grown at 37 °C in 2×YT medium containing 100 μg·mL^−1^ of ampicillin until the culture reached an optical density of 0.4–0.5 at 600 nm and cultivated at 25 °C overnight after the addition of 0.3–0.5 mm β‐d‐isopropylthiogalactopyranoside and 1 mm MnCl_2_. The harvested cells were sonicated in buffer solution (50 mm NaH_2_PO_4_, 300 mm NaCl, pH 8.0), and the produced His‐tagged MetLRE was purified by affinity chromatography using a HisTrap HP column (GE Healthcare Life Sciences, Uppsala, Sweden) and same sonication buffer including imidazole with a 0–500 mm imidazole gradient and dialyzed against 5 mm Tris/HCl, pH 8.0 overnight. The purified C‐terminal His‐tagged MetLRE_his protein solution was used for the enzyme assay and concentrated to 5.5 mg·mL^−1^ using an Amicon Ultra‐15 30 kDa Ultracel (Millipore, Billerica, MA, USA) for crystallization. The purified N‐terminal His‐tagged his_MetLRE and his_MetLRElong2 were also concentrated to 22.3 and 24.9 mg·mL^−1^, respectively, for crystallization, in the same manner as MetLRE_his.

### Enzyme assay

The enzymatic activity of MetLRE_his toward d‐allulose was confirmed as previously measured for an enzyme similar to AgDAE [29]. The reaction mixture consisted of the purified enzyme solution in buffer (50 mm Tris/HCl pH 7.5) containing 1 mm MnCl_2_ and 25 mm d‐allulose. To investigate the optimum temperature of MetLRE_his, the enzyme was incubated at each temperature (30, 40, 50, 60, 70, or 80 °C) for 10 min and its enzymatic activity toward d‐allulose was measured.

To investigate the substrate specificity of MetLRE_his, a reaction mixture consisting of the purified enzyme solution in buffer (50 mm Tris/HCl pH 7.5) containing 1 mm MnCl_2_ and 100 mm substrate (d‐, l‐allulose; d‐, l‐fructose; d‐, l‐tagatose; d‐, l‐sorbose; d‐, l‐xylulose; d‐, l‐ribulose) was incubated at 70 °C for 10 min. After stopping the reaction by boiling for 2 min, the amount of product was measured by HPLC analysis. One unit of enzyme activity was defined as the amount of enzyme that epimerized 1 μmol of substrate per minute [12].

### Molecular weight

The molecular weight of the monomer and molecular size of MetLRE were determined by SDS/PAGE analysis and size exclusion chromatography with a Superdex 200 Increase 10/300 GL column (GE Healthcare Life Sciences), respectively. The molecular size of the native protein was estimated using the Gel Filtration Standard (Bio‐Rad, Hercules, CA, USA) as the standard protein markers with the range of 1.3–670 kDa.

### Thermal stability

A thermal shift assay was carried out using a Viia7 Real‐Time PCR System (Applied Biosystems, Foster City, CA, USA) with SYPRO Orange as the fluorescence tag (Molecular Probes, Eugene, OR, USA) to determine the melting temperature (Tm) of the proteins by detecting the changes in the fluorescence spectra [42, 43].

Briefly, 25 μL of mixture (0.5 mg·mL^−1^ of purified enzyme solution and 5× SYPRO Orange in 40 mm Tris/HCl, pH 7.6, in the presence or absence of 0.8 mm MnCl_2_ and 4 mm d‐allulose) was incubated in a 0.1 mL MicroAmp™ Fast Optical 96‐Well Reaction Plate (Applied Biosystems) at temperatures from 20 °C to 95 °C with a gradient of 0.015 °C increment per 1 s. The assay was performed in duplicate.

The residual activity of MetLRE was measured in the buffer (50 mm Tris/HCl pH 7.5) incubated in the presence and absence of 2 mm MnCl_2_ at 70 °C for different time intervals. The half‐life time (*t*_1/2_) was calculated by measuring the residual activity of MetLRE in the absence of Mn at 60 °C.

### X‐ray crystallography

The crystals of MetLRE_his were grown in a droplet containing 0.8 μL of protein solution (5.5 mg·mL^−1^ in 5 mm Tris/HCl, pH 8.0) and 0.8 μL of reservoir solution [0.2 m MgCl_2_, 0.1 m Tris/HCl pH 8.5, 25% (w/v) PEG3350] against 50 μL of reservoir solution by the sitting‐drop vapor‐diffusion method at 293 K. Each crystal mounted in a cryoloop was soaked in solution containing 15% (w/v) d‐fructose, d‐allulose, or l‐tagatose and flash‐cooled in a stream of nitrogen gas at 100 K.

As for the crystals of his_MetLRE and his_MetLRElong2, each protein solution (his_MetLRE: 22.3 mg·mL^−1^; his_MetLRElong2: 24.9 mg·mL^−1^) in 5 mm Tris/HCl, pH 8.0 and the reservoir solution [0.1 m HEPES pH 7.5, 10% (w/v) polyethylene glycol 8000 and 8% (v/v) ethylene glycol] were used with the above crystallization conditions.

X‐ray diffraction data were collected using the ADSC Quantum 315r CCD detector, PILATUS3 S6M PAD detector on the PF BL5A and the EIGER X 16M PAD detector on the PF BL17A in the KEK (Tsukuba, Japan). Diffraction data were processed using hkl2000 [44], xds [45], and ccp4 program suite [46]. The initial phases of MetLRE_his were obtained by molecular replacement using the program molrep [47, 48] with the structure of AgDAE (PDB code: 5ZFS) as a probe model. Further model building for protein and ligand molecules was performed in the program coot [49], and the structure was refined using the program refmac5 [50, 51]. Data collection and refinement statistics are listed in Table 4. Figures 2, 3, 5, 6, and 7 were drawn using the program pymol (Schrödinger, LLC., New York, NY, USA).

**Table 4:** Data collection and refinement statistics.

	MetLRE	MetLRE/d‐fructose	MetLRE/d‐allulose	MetLRE/l‐tagatose	his_MetLRE/d‐allulose	his_MetLRE_long2/d‐fructose
Data collection						
Beamline	PF BL5A	PF BL5A	PF BL5A	PF BL17A	PF BL5A	PF BL5A
Temperature (K)	100	100	100	100	100	100
Wavelength (Å)	1.000	1.000	1.000	0.980	1.000	1.000
Space group	*P*2_1_2_1_2_1_	*P*2_1_2_1_2_1_	*P*2_1_2_1_2_1_	*P*2_1_2_1_2_1_	*P*1	*P*1
Unit cell parameters (Å)	*a* = 45.73	*a* = 45.42	*a* = 45.75	*a* = 46.06	*a* = 51.02	*a* = 80.43
	*b* = 80.72	*b* = 70.54	*b* = 80.87	*b* = 81.78	*b* = 81.05	*b* = 92.77
	*c* = 139.45	*c* = 140.22	*c* = 139.71	*c* = 140.03	*c* = 106.13	*c* = 93.78
α, β, γ (°)					99.30, 101.50, 87.24	87.67, 98.64, 115.60
Resolution range (Å)	50.00–2.08 (2.13–2.08)	20.00–1.80 (1.85–1.80)	50.00–1.80 (1.85–1.80)	50.00–1.70 (1.73–1.70)	50.00–2.05 (2.10–2.05)	50.00–1.58 (1.62–1.58)
No. of measured reflections	204 455	305 959	305 677	330 615	351 828	1 081 104
No. of unique reflections	31 851 (2299)	42 687 (3104)	48 196 (3464)	58 671 (2875)	100 753 (7341)	311 350 (23 169)
Redundancy	6.4 (6.7)	7.2 (7.3)	6.3 (6.3)	5.6 (6.1)	3.5 (3.6)	3.5 (3.6)
Completeness (%)	99.8 (100.0)	99.9 (100.0)	98.3 (97.0)	98.6 (98.4)	97.6 (96.9)	94.0 (94.7)
Mean *I*_o_/σ(*I*_o_)	13.5 (3.6)	23.2 (3.6)	15.1 (3.9)	17.9 (2.7)	11.5 (2.8)	22.4 (2.8)
*R*_merge_ ^a^ (%)	10.4 (53.5)	6.5 (52.2)	7.7 (46.4)	11.4 (63.5)	8.0 (49.6)	3.4 (45.7)
CC_1/2_	0.998 (0.878)	0.999 (0.884)	0.997 (0.918)	0.991 (0.851)	0.998 (0.835)	0.999 (0.830)
Refinement						
Resolution range (Å)	43.45–2.08 (2.13–2.08)	19.94–1.80 (1.85–1.80)	38.29–1.80 (1.85–1.80)	39.25–1.70 (1.74–1.70)	48.84–2.05 (2.10–2.05)	45.71–1.58 (1.62–1.58)
No. of reflections, working set	30 186 (2173)	40 536 (2925)	45 696 (2439)	55 647 (3826)	95 721 (6926)	295 623 (21 946)
No. of reflections, test set	1605 (120)	2087 (174)	2439 (161)	2961 (212)	5031 (398)	15 718 (1191)
*R*_work_ ^b^ (%)	18.5 (19.5)	12.6 (15.1)	18.1 (22.6)	14.8 (19.0)	16.2 (19.6)	17.7 (18.4)
*R*_free_ ^c^ (%)	26.9 (29.1)	15.3 (19.8)	25.5 (29.3)	18.1 (24.1)	24.1 (31.0)	22.7 (25.2)
R.m.s.d. bond length (Å)	0.004	0.005	0.003	0.005	0.003	0.005
R.m.s.d. bond angles (°)	1.20	1.22	1.24	1.21	1.19	1.25
Ramachandran plot						
Preferred region (%)	94.0	95.9	95.1	95.9	95.4	95.9
Allowed region (%)	5.8	4.1	4.9	3.7	4.5	4.1
B‐factor (Å^2^)						
Protein	27.9	20.7	22.1	15.6	28.7	21.7
Mn	30.7	22.4	21.7	20.9	34.6	20.8
Ligand		24.2 (d‐fructose) 31.5 (d‐fructopyranose)	29.0 (d‐allulose)	22.6 (l‐tagatose) 31.0 (l‐tagatopyranose) 21.0 (l‐sorbose)	36.7 (d‐allulose) 40.9 (HEPES)	26.7 (d‐fructose) 33.0 (HEPES)
Water	33.8	27.5	32.8	21.6	35.3	34.1
PDB ID	7CJ4	7CJ5	7CJ6	7CJ7	7CJ8	7CJ9

Values in parentheses are from the high‐resolution bin.

^a^ *R*_merge_ = Σ*_hkl_* Σ*_i_* [|*I_i_*(*hkl*) − <*I*(*hkl*)>|/Σ*_hkl_* Σ*_i_ I_i_*(*hkl*)], where *I_i_*(*hkl*) is the intensity value of the *i*th measurement of reflection *hkl* and <*I*(*hkl*)> is the mean value of *I_i_*(*hkl*) for all *i* measurements.

^b^ *R*_work_ = Σ*_hkl_*||*F*_obs_| − |*F*_calc_||/Σ*_hkl_*|*F*_obs_|, where *F*_obs_ and *F*_calc_ are the observed and calculated structure factors, respectively.

^c^ *R*_free_ is the free *R*_work_ for the 5% of reflections that were excluded from the refinement.

## Conflict of interest

The authors declare no conflict of interest.

## Ethical approval

This article does not contain any studies with human participants or animals performed by any of the authors.

## Author contributions

HY conceived and designed the experiments, performed cloning, purification, crystallization, and structure analysis; AY performed the characterization of the recombinant enzyme; HY and AY wrote the manuscript; SK and SM contributed to data analysis; KA, KI, and SK contributed to project development and data discussion. All authors discussed the manuscript.

## Data Availability

The atomic coordinates and structure factors of MetLRE alone and in complexes with substrates have been deposited in the Research Collaboratory for Structural Bioinformatics (RCSB) Protein Data Bank (PDB). The PDB codes are 7CJ4 (MetLRE), 7CJ5 (MetLRE/d‐fructose), 7CJ6 (MetLRE/d‐allulose), 7CJ7 (MetLRE/l‐tagatose), 7CJ8 (his_MetLRE/d‐allulose), and 7CJ9 (his_MetLRE_long2/d‐fructose).
